# Effect of multi-enzymes supplementation on growth performance, meat quality, ileal digestibility, digestive enzyme activity and caecal microbiota in broilers fed low-metabolizable energy diet

**DOI:** 10.5713/ab.21.0402

**Published:** 2022-01-21

**Authors:** Muhammad Umar Yaqoob, Muhammad Yousaf, Mubashir Iftikhar, Safdar Hassan, Geng Wang, Safdar Imran, Muhammad Umer Zahid, Waqar Iqbal, Minqi Wang

**Affiliations:** 1 College of Animal Science, Zhejiang University, Key Laboratory of Animal Nutrition and Feed Science (Eastern China), Ministry of Agriculture, Hangzhou 310058, China; 2 Institute of Animal and Dairy Sciences, University of Agriculture, Faisalabad 38000, Pakistan; 3 Five Star Feeds, Pvt. Ltd., Gujranwala 52250, Pakistan; 4 Department of Animal Nutrition, University of Veterinary and Animal Science, Lahore 54000, Pakistan

**Keywords:** Cecal Microflora, Enzyme Activity, Intestinal Morphology, Meat Quality, Multi-enzymes, Nutrient Digestibility

## Abstract

**Objective:**

This study was conducted to evaluate the effect of using low energy diet with multi-enzymes supplementation on different biological parameters in broilers.

**Methods:**

Three hundred Arbor Acres broiler chicks were randomly divided into three groups (Cont, standard metabolizable energy(ME); L-ME, ME reduced by 50 kcal/kg without enzyme; and L-ME-MES, L-ME diet was supplemented with multi-enzymes) with five replicates per group (20 chicks per replicate) at the start of second week. Grower and finisher diets were formulated according to breed specific guide and offered with free access in respective phase (two weeks for grower [8 to 21 d]; two weeks for finisher [22 to 35 d]). External marker method was used to measure the nutrient digestibility. After feeding trial, fifteen birds (one bird per replicate) were selected randomly and slaughtered for samples collection.

**Results:**

The results exhibited no effect (p>0.05) of dietary treatments on all parameters of growth performance, carcass traits, relative weight of internal organs except bursa and overall parameters of thigh meat quality. Relative weight of bursa was significantly (p<0.05) higher in L-ME than control. Multi-enzymes supplementation in low-ME diet significantly (p<0.05) improved the breast meat pH 24 h, digestibility of crude protein, duodenum weight and length, jejunal morphology, counts of *Lactobacillus* spp. and *Bifidobacterium* spp., lipase and protease activities than control. Jejunum length was increased in both L-ME and L-ME-MES treatments than that of the control (p<0.05). Breast meat cooking loss and color lightness was lower in L-ME (p<0.05) than control.

**Conclusion:**

It can therefore be concluded that broilers could be reared on low energy diet with supplementation of multi-enzymes without compromising the growth performance. In addition, it is beneficial for other biological parameters of broilers.

## INTRODUCTION

Exogenous enzymes have been used by animal feed industry for nearly about 4 decades which has significant effect on reducing the cost of production by sparing expensive nutrients [1]. It has been observed that different feed ingredients have some levels of non-starch polysaccharides (NSPs) (cellulose, hemicellulose, arabinan, mannan, and xylan) which could reduce the bioavailability and utilization of nutrients present in the diet [2]. Many researchers proposed the mode of action of these polysaccharide as their anti-nutritional role. Their negative role is reducing the passage rate and enhancing the viscosity of digesta and even decreasing the production of enzymes present in the gastrointestinal tract and not allowing mixing with gut contents [3]. Some other scientists have also observed that the presence of NSPs in the poultry diet can lead to enhanced viscosity of intestinal digesta; decreased digestibility of nutrients; poor feed conversion ratio and bird performance [3]. Non-starch polysaccharides are also present in the feedstuff that are commonly used in the broiler feed formulation and even now there is need to explore the aspects while supplementing the corn-based diets not only with NSPases but also with carbohydrase and proteases. Due to several factors, variations are also present in the common feedstuff. To restrict the effects of variable corn quality and improve nutrient digestibility, exogenous enzymes such as xylanase, amylase and protease are increasingly being used in corn-based diets for broilers [4]. There are several data about the ineffectiveness of xylanase alone because it cannot breakdown the xylan chain due to arabinoses bound to xylans; therefore, an additional enzyme, arabinofuranosidase, is also required for its action to remove first the arabinose unit. Carbohydrate degrading enzymes hydrolyze the components of cell wall like soluble and insoluble arabinoxylans, liberating encapsulated components from inside the cell wall and increasing the contact of endogenous enzymes to the cell contents [5]. Enzymes such as amylase and protease can boost digestive enzymes present inside the body and decrease the losses of endogenous amino acid via changing the production of enzymes from pancreas and secretion of mucin [6]. Products having multi-enzyme activity have been used in broiler diets for 2 decades. Many studies have reported that a combination of xylanase, amylase and protease improve the performance of broilers, energy contents of the feed and degradability of the nutrients in corn-based diets [7]. Recently, Romero et al [8] reported the value of protease supplementation with xylanase and amylase on nutrient digestibility and metabolizable energy (ME). While on other hand, Masey et al [9] reported non-significant results regarding the use of multi-carbohydrase over single enzyme activity. There is scarcity of data showing the advantage of multi-component enzymes at reduced dietary energy level. Therefore, the objective of this study was to examine the effect of exogenous xylanase, amylase and protease on growth performance, meat quality, apparent nutrient digestibility, digestive enzyme activity and caecal microbiota in broilers fed low caloric diet.

## MATERIALS AND METHODS

### Animals and experimental design

The protocol of this study was approved by Institute of Animal and Dairy Sciences, University of Agriculture, Faisalabad, Pakistan for ethical use, care and welfare of experimental animals (IADS/733).

A total of 300 Arbor Acres broiler chicks were used to carry out the feeding trial at R&D farm, Five Star Feeds Pvt. Ltd., Gujranwala, Pakistan. After fed with commercial starter diet (crude protein [CP] 23%; ME 3,000 kcal/kg) for one week, the chicks were randomly divided into three groups (Cont, L-ME, and L-ME-MES: Cont, standard ME without multi-enzymes; L-ME, ME reduced by 50 kcal/kg than standard without multi-enzymes; L-ME-MES, ME reduced by 50 kcal/kg than standard with 100 mg/kg multi-enzymes supplementation) with five replicates per group and 20 chicks per replicate. Axtra XAP 101 (Danisco Animal Nutrition, Marlborough, UK) was used as a source of multi-enzymes with composition of 1,4-β-xylanase 20,000 U/g; α-amylase 2,000 U/g and protease 40,000 U/g. Birds were provided with free access to feed and water throughout the experiment. The formal feeding trial lasted for 4 weeks. The chicks of different groups were fed the grower and finisher diets containing iso-nitrogen and reduced (50 kcal/kg) ME with or without supplementation of multi-enzymes (Table 1). Two phase diets (two weeks for grower, 8 to 21 d; two weeks for finisher, 22 to 35 d) were formulated according to breed specific guide (Arbor Acres broiler nutrition specification, 2019; Table 2).

### Samples collection and measurements

#### Growth performance

Feed intake and body weight were recorded weekly. Collected data were arranged to calculate average weight gain, feed intake and feed conversion ratio (FCR) during each phase (grower and finisher) and on overall basis.

#### Carcass characteristics

After feeding trial, fifteen birds (one bird per replicate) with similar body weight were selected and slaughtered to measure carcass traits. Weight of all carcass parts, abdominal fat and internal organs were recorded and expressed as a percent of carcass weight. Intestinal segments were separated to measure length and weight of each part.

#### Meat quality analysis

Breast and thigh meat samples from each slaughtered bird were also collected for meat quality analysis. Method of Jang et al [10] was followed to determine the water holding capacity (WHC) of breast and thigh meat samples. Samples were centrifuged (Eppendorf Centrifuge5804R, Taufkirchen, Germany) at 5,000 rpm for 10 minutes at 4°C to determine WHC. Meat samples (breast and thigh) were cooked at 90°C for 30 minutes in hot water bath followed by cooling at room temperature, to determine the cooking loss. Initial weight (W1) was recorded and loss in weight after cooking and removing the water was also recorded (W2). Cooking loss was calculated by using following combination:


$$Cooking\;loss\;(\%)=\frac{W1-W2}{W1}\times 100$$

Penetrometry of breast muscles was done to determine the shear force using texture analyzer (Texture Analyser TX-700; LAMY Rheology, Champagne au Mont d’Or, France) in triplicates. Cooked breast muscles were kept perpendicular to the blade and following parameters were set: maximum speed, 1 mm/s; measure time, 10 seconds; force to start, 2 g; wait position, 10 mm; force set, 1,000 g; and maximum distance 50 mm. To determine the pH value of meat sample (breast and thigh) at 24 h, slurry was prepared by homogenizing (OV5; VELP Scientifica, Usmate Velate, Italy) the meat sample (10 g) in distilled water (90 mL) and pH was recorded in duplicate using pH meter (HI 99163; Hanna Instruments Inc., Woonsocket, RI, USA). Meat colorimeter (STPR45; Precision colorimeter, Shenzhen, China) was used to determine the color of breast and thigh meat samples in triplicates. Calibration was done with black and white calibration tiles before using the apparatus according to the manufacturer instructions and CIE L, a, and b values were recorded.

#### Digestibility trial

External marker method was used to determine the nutrient digestibility [11]. External marker, Celite (1%) was added in the last five-day feeds. Ileal contents were collected from slaughtered birds and were immediately packed in plastic bags and stored at −10°C till further analysis, to determine ileal digestibility of the nutrients. Proximate analysis for determination of dry matter (DM), CP, ether extract (EE), and crude fiber (CF) of feed and ileal contents were done following AOAC [12]. Following equation was used for calculation of digestibility coefficient:


$$\text{Digestibility coefficient }(\%)=100-(100\times \frac{Marker\;in\;feed\;(\%)}{Marker\;in\;ileal\;contents\;(\%)}\times \frac{Nutrient\;in\;ileal\;contents\;(\%)}{Nutrient\;in\;feed\;(\%)})$$

#### Digestive enzymes activity

Jejunal digesta was collected from the slaughtered birds and diluted by 4 and 10 times with phosphate-saline buffer followed by centrifugation at 3,000×g for 15 min and at 18,000×g for 20 min at 4°C, respectively. The supernatant was collected and stored at −70°C till further analysis. Activities of lipase, amylase and protease were determined using corresponding kits according to manufacturer’s instructions (Shanghai Changjin Biotechnology Co., Ltd., Shanghai, China). The method of Lowry et al [13] was followed for protein measurement, using bovine serum albumin as standard.

#### Jejunal morphology

Slides of jejunal segments were prepared by dehydrating and then embedding the samples in paraffin followed by sectioning with microtome (5 μm) and stained with hematoxylin and eosin. Morphometry was done by following the previous method [14] with the help of light microscope having computer-assisted morphometric system (Nikon Corporation, Tokyo, Japan).

#### Quantification of cecal microflora

Cecal contents were also collected from slaughtered birds to determine the quantity of different bacteria. Samples were collected and homogenized followed by dilution with sterile 0.9% saline (1:4 wt/vol). Agar plate culture technique was followed for quantification of bacteria. Tenfold dilution of each sample was done and were placed on three replicate plates; for *Lactobacillus* in modified Rogosa, *Bifidobacterium* in TOS Propionate agar, *coliform* in VRBA; *Escherichia coli* (*E. coli*) in MacConkey agar and *Clostridium perfringens* (*C. perfringens*) in tryptose sulphite cycloserine agar. Plates were incubated under anaerobic conditions (except MacConkey agar plates) for 24 to 48 h at 37°C. Following incubation, the bacteria colonies were counted and reported as the log of colony-forming unit per gram (lCFU/g) of digesta.

### Statistical analysis

Data were expressed as the mean±standard error of the mean. All data collected were subjected to one-way analysis of variance using general linear model procedure in SPSS 26. Statistical difference among the means was determined through Tukey’s test (p<0.05).

## RESULTS

### Growth performance

The results exhibited no effect (p>0.05) of dietary treatments on all parameters of growth performance, during each phase or on overall data (Table 3). Weight gain of L-ME-MES group (735.43 g) was reduced than control (746.57 g) during grower phase but FCR was improved by feeding multi-enzymes supplemented low ME diet (1.31 vs 1.33), however the difference was non-significant (p>0.05). During finisher phase weight gain was reduced (p>0.05) in low energy group either supplemented with multi-enzymes or not (L-ME 1,104.84 g; L-ME-MES 1,088.64 g) than control (1,179.83 g), but the efficiency of the bird to convert feed into body weight was numerically better (p>0.05) than control. Similar trend was observed for overall data of growth performance of the birds.

### Carcass characteristics and internal organ indexes

The results exhibited that dietary treatments had no effect (p>0.05) on carcass parameters (Table 4). Numerically better values of relative weight of breast (23.52% vs 22.25%), drumstick (6.77% vs 5.69%), wing (2.27% vs 2.13%) and abdominal fat (3.40% vs 3.10%) were found in L-ME-MES than control. Relative weight of internal organs (heart, liver, gizzard, spleen, and thymus) except, bursa was not affected (p>0.05) by reducing the dietary energy levels with or without supplementation of multi-enzymes (Table 5). In addition, relative weight of bursa was significantly (p<0.05) higher in L-ME than control.

### Meat quality

Data of meat quality showed that some parameters (cooking loss, pH 24 h, and meat color lightness) were influenced (p< 0.05) by dietary treatments only for breast meat. For breast meat, cooking loss (22.57%) and meat color lightness (43.30) in L-ME was lower (p<0.05) than control. In addition, breast meat pH 24 h of L-ME-MES (6.14) was higher (p<0.05) than control (5.90). However, all parameters of thigh meat remained unaffected (p>0.05; Table 6).

### Ileal digestibility

Significant (p<0.05) effect of dietary treatments was found on ileal digestibility of CP. However, digestibility of other nutrients (DM, EE, and CF) remained unaffected. Highest ileal digestibility of CP (74.88%) and CF (15.94%) were found in L-ME-MES. Ileal digestibility of DM and EE were same in all groups (p>0.05; Table 7).

### Digestive enzymes activities

No significant (p>0.05) effect of low ME diet was found on lipase, amylase and protease activity than control group, however multi-enzymes supplementation in low ME diet significantly (p<0.05) improved the activities of lipase (24.93 U/mg) and protease (85.18 U/mg) than control group. On the other hand, amylase activities remained unaffected by dietary treatments (Table 8).

### Intestinal segments and morphology

Significant effects (p<0.05) of dietary treatments were found on the weight and length of some intestinal segments (Table 9). Duodenum weight of L-ME-MES treatment was higher than that of control (p<0.01), however, it was not affected in L-ME. However, weight of other components remained unaffected. As to length of intestinal segments, duodenum (15.94 cm) was increased in L-ME-MES and jejunum length was increased in both L-ME (67.90 cm) and L-ME-MES (67.64 cm), than that of the control (p<0.05). However, remaining components remained unaffected (p>0.05).

Results showed that jejunal morphology was significantly (p>0.05) impaired by reducing the dietary energy levels however, it was improved again by multi-enzymes supplementation in low ME diet (Table 10). Villus height (1,221.60 vs 1,187.60 μm), crypt depth (207.00 vs 202.80 μm) and their ratio (5.90 vs 5.86) was comparable between L-ME-MES and control, respectively.

### Cecal microbiota

Population of beneficial bacteria were improved (p<0.05) by multi-enzymes supplementation in low ME diet, however, counts of pathogenic bacteria were not affected (p>0.05) by dietary treatments (Table 11). Counts of *Lactobacillus* spp. (10.40 lCFU/g) and *Bifidobacterium* spp. (9.40 lCFU/g) were significantly higher in L-ME-MES than that of control.

## DISCUSSION

It has been observed that different feed ingredients have some levels of NSPs which could reduce the availability and utilization of nutrients present in the diet. These NSPs further increase the viscosity of intestinal digesta which negatively affected the nutrients absorption form small intestine. Negative effects of NSPs could be reduced by using multi-enzymes in diets, which was the objective of present study. No significant variations in growth performance was observed by reducing dietary energy levels with or without multi-enzymes supplementation and present observations are supported by previous experiments [13], which stated that reducing dietary ME with or without enzyme supplementation had no influence on the feed intake, weight gain or FCR, which suggested that enzyme supplementation has a critical role in partial replacing the energy from poultry diets. Zhou et al [15] proposed that supplementation of multi-enzymes complex (protease, xylanase, and amylase) in low energy diets enhanced the use of ME in broilers, which could be the possible reason of non-significant difference between control and experimental groups. Gunal et al [16] also stated that supplementation of xylanase or amylase with low energy diet had no significant effect in terms of growth parameters. Sayyazadeh et al [17] checked the effect of exogenous enzyme supplementation by using different dietary energy sources (wheat, barley or its combination) and they get the similar results and found no difference between control and experimental groups for growth related parameters. Present results suggested that multi-enzymes have a role in energy sparing from available ingredients because broilers perform similar at reduced or controlled energy levels with multi-enzymes supplementation. However, Kiarie et al [18] reported that xylanase increased the growth performance in both corn- and wheat-based diets, suggesting the break-down of soluble and insoluble NSPs. Similar findings were reported in corn-soy diets by others [4,19,20] which support the use of xylanases in corn-based diets. Amerah et al [6] reported that positive effects on growth performance in corn-based diets might be due to the high concentration of insoluble arabinoxylan which is present in the corn and being a main component of endosperm cell wall, Xylanase may enhance the access of both endogenous and exogenous enzymes to starch and protein within the endosperm cell.

In the present study, there was hardly any difference noted across the treatments regarding dressing percentage, relative weight of breast, thigh, drumstick and organs (heart, liver, gizzard, spleen, and thymus). However, relative weight of bursa was higher in low-ME diets than other treatments. There are little evidences available in the literature about the influence of multi-enzymes on carcass traits in broilers. No significant results were found regarding carcass traits except gizzard weight in the group consuming high fiber diet. In agreement with our results, Zakaria et al [21] also observed that supplementing broiler’s diets with multi-enzyme had increased the breast meat yield, however, relative weights of other organs were not affected by dietary treatments.

The physical appearance is one of the most vital quality attributes of meat that effect the meat acceptance and purchasing decisions by costumers. All parameters of thigh meat (cooking loss, WHC, pH, color of the meat) remained unaffected (p>0.05) by dietary treatments. For breast meat, cooking loss and meat color lightness in L-ME group was less (p<0.05) than control. However, pH 24 h of L-ME-MES group was higher (p<0.05) than control. Color of the breast meat was significantly improved in our study however; Zakaria et al [21] reported that the dietary enzymes supplementation did not affect meat quality parameters in broilers. Different meat quality parameters are interlinked to each other, such as meat color and pH value, which are mainly affected by concentration of hemoglobin.

Significantly (p <0.05) highest ileal digestibility of CP was observed in L-ME-MES. However, ileal digestibility of other nutrients remained unaffected (p>0.05) by dietary treatments. Our results are in consistent with the findings of Amerah et al [6] which stated that combination of xylanase, amylase and protease improved the digestibility of nitrogen in broilers. The effect of protease on nutrient digestibility in broilers, however, has been varying but Amerah et al [6] reported 2.2% more N digestibility when protease was combined with xylanase and amylase than with protease alone. Similar results were reported by Romero et al [8], which suggested that supplementation of multi-enzymes as combination (xylanase, amylase and protease) improved the protein digestibility. Supplementation of multi-enzymes in low ME diet significantly (p<0.05) improved the activities of lipase and protease than control group. On the other hand, amylase activities remained unaffected by dietary treatments. Similar results were observed by Yuan et al [22] reported that amylase and lipase activities were significantly higher (p<0.05) in NSPase supplemented animals than those without NSPase. The amount of digestive juice secreted by different organs like liver, pancreas and intestinal mucosa and the activity of enzymes determine digestive function. Similarly, Engberg et al [23] also reported that xylanase supplementation significantly enhanced the chymotrypsin and lipase activity in broilers. However, Mirzaie et al [24] reported no effect of supplementing wheat-based diets with xylanase but exogenous enzyme reduced the viscosity of the intestine. According to another study, diets based on ingredients rich in NSPs such as maize, barley, wheat and sorghum affected intestinal enzymes activity, while enzyme supplementation did not [25]. Increased protease activities in multi-enzyme supplemented group also supported the results of increased protein digestibility in this study.

Duodenum weight and length in L-ME-MES treatment was higher than that of control (p<0.01). Jejunum length was increased in both L-ME and L-ME-MES treatments than that of the control (p<0.05). Results showed that jejunal morphology was significantly (p>0.05) impaired by reducing the dietary energy levels however, it was improved again by multi-enzymes supplementation in low ME diet. Villus height, crypt depth and their ratio were comparable between L-ME-MES and control, respectively. For nutrient absorption, small intestine is a main part of the digestive tract. Columnar cells of the intestine can differentiate into mucin producing, digestive and absorptive roles [20]. Intestinal morphological parameters such as crypt depth, villus height and goblet cell are affected by dietary manipulation. Hussain et al [20] reported no improvements in intestinal morphology by the supplementation of a blend of exogenous enzymes including xylanase, protease and mannanase. They reported that weight and length of all parts of small intestine (duodenum, jejunum and ileum) remained unaffected by the enzyme even when they supplemented alone or in combination. Similar results were found by Opoku et al [26] when they supplemented broilers diet with protease and mannase. Poor nutrient absorption could be due to shorter villus and deeper crypts resulting in increased secretion of water and electrolytes in gastrointestinal tract and thus compromising performance.

Counts of pathogenic bacteria were not affected (p >0.05) by dietary treatments, however, *Lactobacillus* spp. and *Bifidobacterium* spp. were significantly increased in L-ME-MES than that of control. In the agreement with present study, Shakouri et al [25] reported higher cecal bacterial (*Enterobacteriaceae*) count in broilers fed semi purified diet supplemented with NSPase. Similarly, Yaghobfar and Kalantar [27] reported that the beneficial bacteria including lactic acid bacteria and *bifidobacteria* were lower in the diets not containing NSPases and higher in the birds fed diets supplemented with NSPases. However, pathogenic bacteria including *E. coli* and *clostridia* were also significantly higher in the birds fed on wheat or barley-based diets, without supplementation of enzymes. Dersjant et al [28] used multi-enzyme supplementation in broiler’s diet and found that there was a tendency of reducing *C. perfringens* population in cecal digesta. It may be speculated that multi-enzymes supplementation might have a positive effect on microflora in the intestine, by lowering pathogenic bacteria such as *C. perfringens*. These outcomes have the tendency to improve the intestinal health of birds.

## CONCLUSION

It can therefore be concluded that supplementation of combination of xylanase, amylase and protease in low-ME broiler diets could be helpful to improve the nutrient digestibility, intestinal morphology, count of beneficial bacteria and enzyme activities without compromising the growth performance and carcass traits in broilers.

## Figures and Tables

**Table 1:** Experimental plan

Parameters	Treatments^1)^		

	Control	L-ME	L-ME-MES
NSPase^2)^	0.00	0.00	100 mg/kg
Metabolizable energy (kcal/kg)			
Grower diet	3,100	3,050	3,050
Finisher diet	3,200	3,150	3,150

1) Control, standard ME without multi-enzymes; L-ME, metabolizable energy reduced by 50 kcal/kg than standard without multi-enzymes; L-ME-MES, metabolizable energy reduced by 50 kcal/kg than standard with multi-enzymes supplementation.

2) NSPase: Axtra XAP 101: 1,4-β-xylanase 20,000 U/g; α-amylase 2,000 U/g; Protease 40,000 U/g (Danisco Animal Nutrition, Marlborough, UK).

**Table 2:** Ingredient and chemical composition of diets

Items	Treatments^1)^			

	Grower (8 to 21 d)		Finisher (22 to 35 d)	
	
	Cont	L-ME	Cont	L-ME
Ingredients				
Corn (%)	55.67	56.83	62.27	63.48
Soya bean meal (%)	27.79	27.65	23.56	23.56
Rapeseed meal (%)	8.00	8.68	6.09	6.63
Veg. oil (%)	5.04	3.32	5.18	3.42
Limestone (%)	1.24	1.24	1.03	1.04
Monocalcium phosphate (%)	0.74	0.75	0.51	0.51
L-Lysine HCl (%)	0.38	0.39	0.34	0.34
DL-Methionine (%)	0.27	0.27	0.23	0.23
Threonine (%)	0.08	0.08	0.06	0.06
NaCl (%)	0.25	0.25	0.22	0.22
Sodium bicarbonate (%)	0.23	0.23	0.20	0.20
Phytase (%)	0.01	0.01	0.01	0.01
VitaMin premix^2)^ (%)	0.30	0.30	0.30	0.30
Total	100	100	100	100
Chemical composition				
Metabolizable energy (kcal/kg)	3,100	3,050	3,200	3,150
Crude protein (%)	21.50		19.50	
Lysine (%)	1.29		1.16	
Methionine (%)	0.51		0.47	
Calcium (%)	0.87		0.79	
Available P (%)	0.44		0.40	

1) Cont (Control), standard metabolizable energy without multi-enzymes; L-ME, metabolizable energy reduced by 50 kcal/kg than standard without multi-enzymes.

2) Vit. A 15,000 IU/kg, D_3_ 3,000 IU/kg, E 60 IU/kg, K_3_ 3 mg/kg, B_1_ 2 mg/kg, B_2_ 8 mg/kg, niacin 45 mg/kg, pantothenic acid 15 mg/kg, B_6_ 4 mg/kg, folic acid 1 mg/kg, B_12_ 1 mg/kg, choline chloride (60%) 500 mg/kg, magnesium sulphate 53 mg/kg, manganese sulphate 18.5 mg/kg, zinc sulphate 2 mg/kg, ferrous sulphate 35 mg/kg and copper sulphate 45 mg/kg.

**Table 3:** Effect of multi-enzymes supplementation on growth performance in broilers fed low caloric diet

Parameters	Treatments^1)^		

	Control	L-ME	L-ME-MES
Grower (2–3 weeks)			
Feed intake (g)	987.75±51.50	1,018.12±24.56	966.62±30.27
Weight gain (g)	746.57±15.99	750.29±12.98	735.43±13.70
FCR (g/g)	1.33±0.08	1.36±0.05	1.31±0.02
Finisher (4–5 weeks)			
Feed intake (g)	2,178.96±74.60	2,102.33±35	2,024.68±43.14
Weight gain (g)	1,179.83±31.37	1,104.84±28.03	1,088.64±23.07
FCR (g/g)	1.85±0.09	1.90±0.02	1.86±0.04
Overall (2–5 weeks)			
Feed intake (g)	3,166.72±56.47	3,120.45±29.01	2,991.29±68.28
Weight gain (g)	1,906.40±53.42	1,995.13±43.88	1,878.07±47.62
FCR (g/g)	1.67±0.08	1.57±0.02	1.59±0.03

Data are presented as mean±standard error of the mean.

FCR, feed conversion ratio.

1) Cont, standard metabolizable energy without multi-enzymes; L-ME, metabolizable energy reduced by 50 kcal/kg than standard without multi-enzymes; L-ME-MES, metabolizable energy reduced by 50 kcal/kg than standard with multi-enzymes supplementation.

**Table 4:** Effect of multi-enzymes supplementation on carcass parts in broilers fed low caloric diet

Items	Treatments^1)^		

	Control	L-ME	L-ME-MES
Dressing percentage (%)	68.13±2.39	65.62±1.98	67.98±1.46
Breast (%)^2)^	22.25±0.90	21.89±0.20	23.52±1.09
Thigh (%)^2)^	19.93±1.51	18.51±0.82	18.28±1.14
Drumstick (%)^2)^	5.69±0.80	5.30±0.67	6.77±0.48
Wing (%)^2)^	2.13±0.16	2.47±0.08	2.27±0.47
Abdominal fat (%)^2)^	3.10±0.40	3.42±0.44	3.40±0.47

Data are presented as mean±standard error of the mean.

1) Control, standard metabolizable energy without multi-enzymes; L-ME, metabolizable energy reduced by 50 kcal/kg than standard without multi-enzymes; L-ME-MES, metabolizable energy reduced by 50 kcal/kg than standard with multi-enzymes supplementation.

2) Percent of carcass weight.

**Table 5:** Effect of multi-enzymes supplementation on organs indexes in broilers fed low caloric diet

Parameters (% of carcass weight)	Treatments^1)^		

	Control	L-ME	L-ME-MES
Heart	1.25±0.16	1.31±0.07	1.25±0.04
Liver	3.74±0.17	4.12±0.19	4.10±0.07
Gizzard	3.81±0.23	4.33±0.26	4.18±0.38
Bursa	0.45±0.01	0.65±0.05^*^	0.54±0.02
Spleen	0.29±0.02	0.40±0.04	0.33±0.03
Thymus	0.77±0.05	0.95±0.07	0.92±0.05

Data are presented as mean±standard error of the mean.

1) Control, standard metabolizable energy without multi-enzymes; L-ME, metabolizable energy reduced by 50 kcal/kg than standard without multi-enzymes; L-ME-MES, metabolizable energy reduced by 50 kcal/kg than standard with multi-enzymes supplementation.

Statistic difference from corresponding control value for a given parameter is annotated by:

* p<0.05.

**Table 6:** Effect of multi-enzymes supplementation on meat quality traits in broilers fed low caloric diet

Parameters	Treatments^1)^		

	Control	L-ME	L-ME-MES
Breast meat			
Shear force (g)	1,025.79±7.30	1,049.48±12.97	1,051.18±15.28
Cook loss (%)	26.38±1.36	22.57±0.66^*^	27.03±0.61
WHC (%)	44.75±1.63	44.08±0.77	46.14±0.66
pH at 24 h	5.90±0.07	6.07±0.03	6.14±0.03^*^
L	51.58±1.35	43.30±2.38^*^	49.17±1.36
a	10.03±1.71	9.30±0.88	7.72±0.41
b	13.58±0.53	11.56±0.82	12.08±0.45
Thigh meat			
Cook loss (%)	31.91±0.53	32.33±1.37	31.08±0.39
WHC (%)	41.82±2.98	41.49±0.76	42.47±1.31
pH at 24 h	6.07±0.01	6.22±0.16	6.27±0.02
L	53.37±2.32	52.42±2.74	52.91±2.03
a	6.74±0.97	7.33±0.52	5.98±0.58
b	7.65±1.21	8.74±1.56	9.74±1.39

Data are presented as mean±standard error of the mean.

WHC, water holding capacity.

1) Control, standard metabolizable energy without multi-enzymes; L-ME, metabolizable energy reduced by 50 kcal/kg than standard without multi-enzymes; L-ME-MES, metabolizable energy reduced by 50 kcal/kg than standard with multi-enzymes supplementation.

Statistic difference from corresponding control value for a given parameter is annotated by:

* p<0.05.

**Table 7:** Effect of multi-enzymes supplementation on ileal digestibility of nutrients in broilers fed low caloric diet

Parameters (%)	Treatments^1)^		

	Control	L-ME	L-ME-MES
Dry matter	73.69±0.20	73.81±0.68	73.26±0.43
Crude protein	70.15±0.63	69.74±0.33	74.88±0.98^*^
Ether extract	72.95±0.76	70.34±1.95	71.11±1.35
Crude fiber	14.45±0.90	12.68±0.97	15.94±0.42

Data are presented as mean±standard error of the mean.

1) Control, standard metabolizable energy without multi-enzymes; L-ME, metabolizable energy reduced by 50 kcal/kg than standard without multi-enzymes; L-ME-MES, metabolizable energy reduced by 50 kcal/kg than standard with multi-enzymes supplementation.

Statistic difference from corresponding control value for a given parameter is annotated by:

* p<0.05.

**Table 8:** Effect of multi-enzymes supplementation on jejunal digestive enzymes activities in broilers fed low caloric diet

Parameters (U/mg)	Treatments^1)^		

	Control	L-ME	L-ME-MES
Lipase	22.38±0.66	20.88±0.55	24.93±0.48^*^
Amylase	13.53±0.95	14.27±1.03	16.87±0.64
Protease	76.36±0.58	78.01±0.35	85.18±0.53^*^

Data are presented as mean±standard error of the mean.

1) Control, standard metabolizable energy without multi-enzymes; L-ME, metabolizable energy reduced by 50 kcal/kg than standard without multi-enzymes; L-ME-MES, metabolizable energy reduced by 50 kcal/kg than standard with multi-enzymes supplementation.

Statistic difference from corresponding control value for a given parameter is annotated by:

* p<0.05.

**Table 9:** Effect of multi-enzymes supplementation on intestinal segments in broilers fed low caloric diet

Parameters	Treatments^1)^		

	Control	L-ME	L-ME-MES
Weight (g)			
Duodenum	20.84±0.22	20.90±0.24	22.70±0.36^*^
Jejunum	32.12±1.91	32.00±1.26	31.80±1.56
Ileum	24.42±0.98	23.34±0.55	24.60±1.33
Ceca	11.78±0.37	11.62±0.25	11.04±0.45
Length (cm)			
Duodenum	14.28±0.46	15.05±0.17	15.94±0.33^*^
Jejunum	62.66±0.29	67.90±1.14^*^	67.64±0.61^*^
Ileum	64.24±1.22	61.63±1.31	62.06±1.74
Ceca	17.86±1.03	16.60±0.93	15.70±0.44

Data are presented as mean±standard error of the mean.

1) Control, standard metabolizable energy without multi-enzymes; L-ME, metabolizable energy reduced by 50 kcal/kg than standard without multi-enzymes; L-ME-MES, metabolizable energy reduced by 50 kcal/kg than standard with multi-enzymes supplementation.

Statistic difference from corresponding control value for a given parameter is annotated by:

* p<0.05.

**Table 10:** Effect of multi-enzymes supplementation on jejunal morphology in broilers fed low caloric diet

Parameters	Treatments^1)^		

	Control	L-ME	L-ME-MES
Villus height (μm)	1,187.60±25.70	944.00±19.13^*^	1,221.60±12.19
Crypt depth (μm)	202.80±4.93	254.80±8.40^*^	207.00±2.00
Villus/crypt	5.86±0.03	3.71±0.07^*^	5.90±0.03

Data are presented as mean±standard error of the mean.

1) Control, standard metabolizable energy without multi-enzymes; L-ME, metabolizable energy reduced by 50 kcal/kg than standard without multi-enzymes; L-ME-MES, metabolizable energy reduced by 50 kcal/kg than standard with multi-enzymes supplementation.

Statistic difference from corresponding control value for a given parameter is annotated by:

* p<0.05.

**Table 11:** Effect of multi-enzymes supplementation on cecal microbiota in broilers fed low caloric diet

Parameters (lCFU/g)	Treatments^1)^		

	Control	L-ME	L-ME-MES
*Lactobacillus* spp.	8.40±0.24	9.20±0.20	10.40±0.24^*^
*Bifidobacterium* spp.	7.40±0.24	8.40±0.24	9.40±0.24^*^
*Clostridium perfringens*	6.40±0.68	6.20±0.80	6.20±0.73
*Escherichia coli*	6.60±1.03	5.80±0.66	5.60±0.81

Data are presented as mean±standard error of the mean.

lCFU, log of colony-forming unit.

1) Control, standard metabolizable energy without multi-enzymes; L-ME, metabolizable energy reduced by 50 kcal/kg than standard without multi-enzymes; L-ME-MES, metabolizable energy reduced by 50 kcal/kg than standard with multi-enzymes supplementation.

Statistic difference from corresponding control value for a given parameter is annotated by:

* p<0.05.
